# Disseminated Varicella-Zoster Virus Infection with Internal Organ Involvement: A Scoping Review of 156 Cases

**DOI:** 10.3390/v17081135

**Published:** 2025-08-19

**Authors:** Aleksandar Timotijevic, Pratyusha Kodela, Vladislav Glušac, Sara Bokonjic, Bojan Joksimovic, Juan Vera Gomez, David Ladin, Igor Dumic

**Affiliations:** 1 Department of Hospital Medicine, Mayo Clinic Health System, Eau Claire, WI 54703, USA; aleksandar.timotijevic@franciscanalliance.org (A.T.); pratyushakodela@gmail.com (P.K.); veragomez.juan@mayo.edu (J.V.G.); ladin.david@mayo.edu (D.L.); 2 Internal Medicine Residency Program, Franciscan Health Olympia Fields Hospital, Olympia Fields, IL 60461, USA; 3 School of Medicine, University of Belgrade, 11000 Belgrade, Serbia; vladaglu@gmail.com; 4 Institute of Diagnostic and Interventional Radiology, Medical Faculty and University Hospital, Heinrich Heine University, 40225 Dusseldorf, Germany; sbokonjic@gmail.com; 5 Department of Pathological Physiology, Faculty of Medicine, University of East Sarajevo, 73300 Foca, Bosnia and Herzegovina; bojannjoksimovic@gmail.com; 6 Department of Pediatrics, ASA Hospital, 71000 Sarajevo, Bosnia and Herzegovina; 7 Mayo Clinic College of Medicine and Science, Rochester, MN 55905, USA

**Keywords:** varicella-zoster virus, visceral involvement, varicella pneumonia, varicella hepatitis, varicella myocarditis, visceral VZV infection, VZV pancreatitis, splenomegaly

## Abstract

Visceral disseminated varicella-zoster virus infection (VD-VZV) involves the hematogenous spread of VZV from the skin to the internal organs. Though rare, it is potentially life-threatening, predominantly affecting immunocompromised individuals. Diagnosis is often delayed due to nonspecific symptoms mimicking other viral illnesses. While the vesicular rash is a hallmark sign, it is absent in approximately 5% of cases. Visceral involvement may precede cutaneous lesions, complicate early recognition, and increase the risk of severe complications. This scoping review screened 594 articles of which 153 met the inclusion criteria, yielding 156 individual cases. Patients were predominantly male (53.8%), with a mean age of 42.3 years. The overall mortality rate was 25.0%. Multiple organs were involved in 46.1% of cases. The most frequently affected were the lungs (56%), liver (44%), heart (16%), kidneys (11%), pancreas (11%), stomach (10%), and esophagus (6%). Antivirals were administered in 89.1% of cases, while corticosteroids were used in 22.4%, with no significant impact on outcomes. Early diagnosis, achieved in 65.4% of patients, was significantly associated with survival (*p* = 0.043). Mortality was significantly associated with underlying comorbidities (*p* = 0.004), especially autoimmune diseases requiring immunosuppression (*p* = 0.048). Septic shock or multi-organ dysfunction (MODS), hepatitis, acute kidney injury, and acute liver failure were linked to higher mortality in univariate analysis. Multivariate analysis identified comorbidities (*p* < 0.001), septic shock/MODS (*p* = 0.008), and acute liver failure (*p* = 0.039) as independent predictors of mortality. Patients with septic shock/MODS had over twice the risk of death (OR = 2.24; *p* = 0.008). This review underscores the diagnostic challenges and high mortality of VD-VZV. Early recognition and timely administration of antiviral treatment appear critical for survival. Greater clinical awareness and further research are needed to guide management.

## 1. Introduction

The varicella-zoster virus (VZV), a member of the *Orthoherpesviridae* family, is a medically important human pathogen responsible for two primary clinical syndromes: varicella (chickenpox), typically occurring in childhood, and herpes zoster (shingles), which presents later in life as a result of viral reactivation [1]. In immunocompetent individuals, the primary VZV infection is generally benign and self-limiting. However, adults experience complications more frequently, and both pediatric and adult immunocompromised populations are at increased risk for disseminated disease, which is associated with significant morbidity and mortality [2]. In most cases, primary VZV infection manifests as a vesiculopustular rash accompanied by fever, resolving within approximately two weeks. Disseminated varicella is distinguished by a more extensive eruption, often involving mucous membranes, internal (visceral) organs, and multiple dermatomes, in addition to systemic symptoms, such as fever and malaise [1,2,3].

VZV is a strictly human pathogen with no known animal reservoir and demonstrates high transmissibility [1,2,3,4]. Transmission occurs primarily through airborne respiratory droplets and direct contact with vesicular fluid from infected skin lesions [4]. The virus exhibits tropism for T lymphocytes, epithelial cells, and sensory ganglia. During primary infection, viremia facilitates widespread cutaneous involvement [1,2,3,4,5]. The innate immune system detects VZV through pattern recognition receptors, including Toll-like receptors (TLRs) [5,6,7,8,9]. Despite eliciting an immune response, VZV evades complete clearance by subverting immune mechanisms and altering host cell function [5,6,7,8,9]. It establishes lifelong latency in the sensory neurons of the dorsal root and trigeminal ganglia, with potential for reactivation. Herpes zoster, the clinical manifestation of reactivation, presents as a painful, dermatomally distributed vesicular rash and occurs more frequently with advancing age due to immunosenescence, as well as in individuals with immunocompromised status [5,6,7,8,9].

Disseminated VZV infection is an acute, severe, and life-threatening condition that occurs predominantly in immunocompromised individuals. Although far less common than uncomplicated varicella, disseminated infection is associated with substantially higher risks of complications and mortality [10,11,12]. Visceral disseminated VZV (VD-VZV) refers to hematogenous dissemination of the virus into internal organs, resulting in multi-organ involvement [2,10,11,12,13]. Clinical diagnosis is often challenging, as the disease mimics other serious conditions, such as other viral pneumonia, hepatitis, pancreatitis, myocarditis, nephritis, and esophagitis [2,13]. Diagnostic uncertainty is further compounded in a minority of patients who do not exhibit the characteristic cutaneous rash. While VD-VZV is primarily observed in profoundly immunosuppressed individuals, it has also been reported in immunocompetent hosts [13,14]. Due to its rarity, the true incidence remains unknown, and existing knowledge is largely based on isolated case reports and small case series, without any large-scale prospective or retrospective studies.

The primary aim of this scoping review is to synthesize data from published case reports and case series to characterize demographics, risk factors, commonly affected internal organs, treatment and clinical outcomes in patients diagnosed with VD-VZV. Given the infrequency of this condition and the practical challenges in conducting prospective studies, this review seeks to consolidate existing evidence to enhance clinical recognition, improve diagnostic accuracy, and identify patterns that may support therapeutic decision-making. By delineating these aspects, the review aims to raise clinical awareness, inform medical practice, and stimulate further research to advance the understanding, diagnosis, and management of visceral VZV infection.

## 2. Materials and Methods

### Search Strategy, Definitions, and Selection Criteria

**Figure 1:**
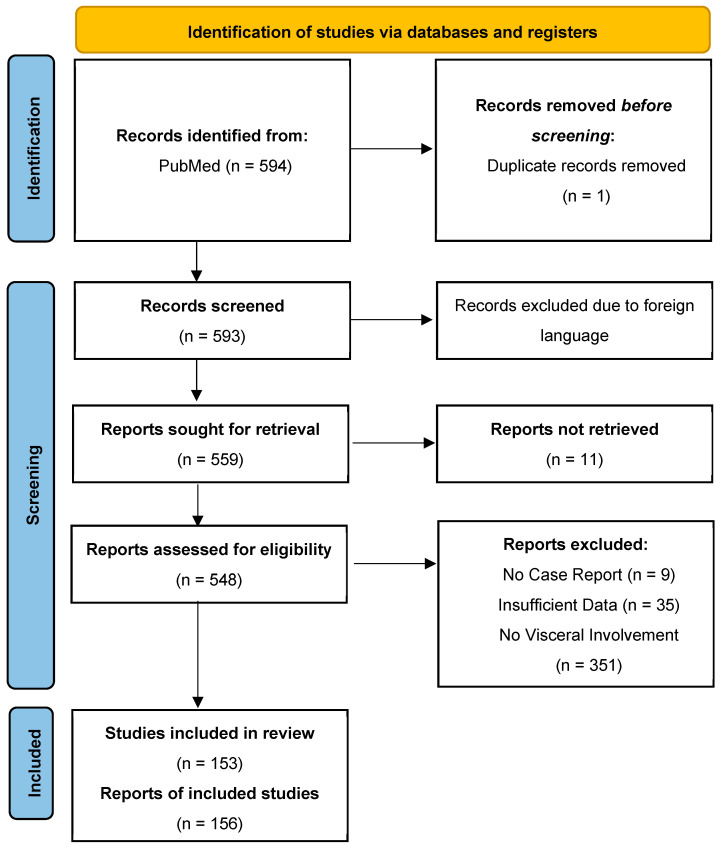
Prisma flowchart used for the literature review and final article selection included in the analysis.

This scoping review was conducted in accordance with the Preferred Reporting Items for Systematic Reviews and Meta-Analyses (PRISMA) guidelines (Figure 1) using Medline (National Library of Medicine, Bethesda, MD, USA) via the PubMed search engine from 1 July 2004 to 1 March 2024. All included cases had a documented visceral presentation of VZV infection. The timeframe after the year 2004 used in this review was chosen to reflect the diagnostic and therapeutic modalities used today, making any statistically significant findings more likely to be relevant today.

A total of 594 original articles from Medline were identified that mention a combination of MeSH and non-MeSH terms, “disseminated varicella zoster” OR “visceral varicella zoster” OR “visceral herpes virus 3” OR “disseminated visceral varicella” OR disseminated visceral zoster” OR “varicella pneumonia” OR varicella pneumonitis” OR varicella hepatitis” OR varicella myocarditis” OR “varicella pericarditis” OR “varicella myopericarditis” OR “varicella gastritis”. The following search filters were used: ‘case reports’, ‘humans’, and period ‘1 July 2004 to 1 March 2024’. In our study, we included both pediatric and adult patients.

We included cases with a proven VZV infection based on clinical presentation and confirmatory PCR (on blood, tissues, or vesicular fluid sample), tissue biopsy, blood serology (IgM against VZV), or autopsy findings with clearly documented visceral organ involvement. Visceral organ involvement during varicella-zoster virus (VZV) infection was defined as the presence of acute pathological findings in internal organs occurring during the course of infection. Neurological manifestations have been excluded as they represent distinct clinical syndromes.

We reviewed clinical signs and symptoms, physical examination findings, and systematically collected data for each organ or organ system. Inclusive decisions were based on the following diagnostic criteria:

- Lungs: Involvement was defined by clinical and radiographic evidence of pneumonia or pneumonitis in an adequate clinical setting. Diagnostic tools included PCR testing of bronchoalveolar lavage specimens, chest radiography (CXR), and computed tomography (CT) of the thorax.
- Liver: Involvement was identified based on biochemical and histological findings of hepatitis. Laboratory abnormalities included elevated levels of aspartate aminotransferase (AST), alanine aminotransferase (ALT), alkaline phosphatase (ALP), and bilirubin; and histopathological confirmation via liver biopsy (when available) was considered. Acute liver failure (ALF) diagnosis was based on markedly increased AST and ALP, International Normalized Ratio (INR) ≥ 1.5, and encephalopathy in the absence of pre-existing hepatic disease.
- Kidneys: Renal dysfunction was determined by elevated serum creatinine levels, decreased glomerular filtration rate (GFR), oliguria and/or anuria, and, where available, a kidney biopsy.
- Heart: Cardiac involvement was diagnosed based on findings of myocardial injury with elevated serum cardiac biomarkers (troponin), transthoracic echocardiography (TTE), cardiac magnetic resonance imaging (MRI), and, where available, a heart muscle biopsy demonstrating features of myocarditis.
- Gastrointestinal System: Gastrointestinal involvement included abnormalities in the esophagus, stomach, and intestines. Diagnostic modalities included were esophagogastroduodenoscopy (EGD), colonoscopy, and biopsy findings.
- Pancreas: Diagnosis of pancreatitis was based on elevated levels of lipase—more than 3 times the upper normal limit, CT of the abdomen demonstrating changes consistent with pancreatitis, MRI findings, and, when available, a biopsy.
- Spleen: Splenic involvement was determined based on abdominal ultrasound and CT of the abdomen, demonstrating pathological changes within the spleen.

In patients with pre-existing chronic medical conditions, we included only the cases where VZV infection led to clear exacerbation of a pre-existing disease in an appropriate clinical setting and consistent timeline related to VZV infection.

We excluded cases where the diagnosis was uncertain either because laboratory confirmation, biopsy, or autopsy was not performed, or visceral organ biopsy was negative. We also excluded duplicate articles, articles in languages other than English, abstracts without comprehensive case descriptions, and narrative reviews. Ultimately, our review included 156 individual cases that fulfilled the inclusion criteria. Figure 1 shows the detailed process of article selection and the final cases included in the analysis.

All case reports were manually screened by the first author (AT); the second author (PK) and the senior author (ID) provided clarification where needed before the final selection of cases. We also used the Rayyan software as a tool, a web-based application developed by QCRI (Qatar Computing Research Institute, Doha, Qatar) that helps guide the process of screening and removal of duplicates.

The following data were extracted from all cases and documented in an Excel table: demographic characteristics, comorbidities, length of hospitalization, presence of skin rash, abdominal pain, organs involved, evidence of multiple visceral involvement, occurrence of multiorgan dysfunction, septic shock, or fulfillment of SIRS criteria, serologic testing for VZV, vaccination status, imaging findings, laboratory results, timeliness of diagnosis, treatment administered, and clinical outcomes. In cases where specific information was unavailable, “not reported” was entered in the corresponding field.

Descriptive statistics methods included measures of central tendency and measures of variability, specifically the following: arithmetic mean with standard deviation (M ± SD), median, and interquartile range (Mdn/IQR), as well as relative frequencies for categorical variables. Before statistical data analysis, the normality of the distribution of obtained values was examined using the Shapiro–Wilk test. In the case of normal distribution, a parametric *t*-test for independent samples was used to compare the average values of variables between two populations. When the distribution was not normal, the non-parametric Mann–Whitney test was used. Among non-parametric tests, the chi-square test was used to observe differences in the frequencies of certain occurrences between groups. A binary logistic regression model was used for multivariate analysis to assess the association between mortality in patients with VD-VZV infection and multiple variables. A statistically significant difference between the values obtained from the groups was considered at *p* < 0.050. For statistical data analysis, the software package SPSS version 21.0 (“Statistical Package for Social Sciences SPSS 21.0 Inc., New York, NY, USA”) was used.

## 3. Results

### 3.1. Literature Search

Our initial search of the Medline database over 20 years yielded 594 records, of which 1 was a duplicate. We screened and assessed the titles and abstracts of all 593 non-duplicate records, excluding 440 records that did not meet the inclusion criteria. A total of 153 articles yielded 156 cases that fulfilled the inclusion criteria for analysis [15,16,17,18,19,20,21,22,23,24,25,26,27,28,29,30,31,32,33,34,35,36,37,38,39,40,41,42,43,44,45,46,47,48,49,50,51,52,53,54,55,56,57,58,59,60,61,62,63,64,65,66,67,68,69,70,71,72,73,74,75,76,77,78,79,80,81,82,83,84,85,86,87,88,89,90,91,92,93,94,95,96,97,98,99,100,101,102,103,104,105,106,107,108,109,110,111,112,113,114,115,116,117,118,119,120,121,122,123,124,125,126,127,128,129,130,131,132,133,134,135,136,137,138,139,140,141,142,143,144,145,146,147,148,149,150,151,152,153,154,155,156,157,158,159,160,161,162,163,164,165,166,167,168,169,170,171,172,173,174,175,176].

Figure 1 illustrates the flow chart with details of articles selected and final studies included.

### 3.2. Demographics and Comorbidities

We included 156 patients, of whom 84 (53.8%) were male, with a mean age of 42.32 ± 20.77 years, of whom 21 were patients under 18 years of age (13.6%). The mortality rate was 25.0% (*n* = 39). Patients who died more often had comorbidities (*p* = 0.004), especially autoimmune diseases on immunosuppressive therapy (IST) (*p* = 0.048), compared to those who survived. The mean hospitalization length was 22.09 ± 28.50 days, without difference relative to survival (Table 1).

### 3.3. Visceral (Internal) Organ Involvement

Seventy-two patients (46.1%) had multiple visceral organ involvement. As shown in Table 1, the most commonly affected internal organs in disseminated VZV were the lungs (56%), liver (44%), heart (16%), kidneys (11%), pancreas (11%), stomach (10%), and esophagus (6%). Other organs were involved in less than 5% of cases.

Univariate analyses demonstrated that patients who died had a significantly higher frequency of septic shock/multiple organ dysfunction (*p* < 0.001), hepatitis (*p* = 0.032), acute kidney injury/kidney failure (*p* = 0.049), and acute liver failure (*p* < 0.001).

### 3.4. Diagnostic Workup and Treatment

A positive PCR for VZV in blood, organ tissue, or vesicular fluid was detected in 75 patients, representing 48% of our cohort. Based on a combination of positive biopsy findings, positive PCR results on clinical specimens, and the presence of VZV-specific IgM antibodies, all patients were diagnosed with definitive acute VZV infection (Table 1). Patients who died were more likely to have positive findings on lung biopsies (*p* = 0.012) and liver biopsies (*p* = 0.029). Bronchoalveolar lavage (BAL) PCR was conducted in 15 patients (9.6%), all of whom had positive findings (100%). Laboratory analyses showed significantly higher median values of AST (*p* < 0.001), ALT (*p* < 0.001), and INR (*p* = 0.004) in patients who died compared to survivors. Survivors were more likely to have positive VZV IgG antibodies (*p* = 0.028).

Chest CT scans were performed in 47 patients (30.1%). Among these, pleural effusion (*p* = 0.034) and nodular infiltrates (*p* = 0.019) were significantly more common in patients who died. Heart MRIs were performed in seven patients (4.5%), with positive findings observed in six cases (85.7%).

A timely diagnosis was established in 102 patients (65.4%), and survivors were significantly more likely to receive a timely diagnosis compared to those who died (*p* = 0.043). The majority of patients (*n* = 139, 89.1%) received antiviral therapy. Corticosteroids were used in combination with antivirals in 35 patients (22.4%), but this combination did not influence infection outcomes (Table 1).

Multivariate analysis showed that independent risk factors for mortality were the presence of comorbidities (*p* < 0.001), septic shock/multiple organ dysfunction (*p* = 0.008), and acute liver failure (*p* = 0.039). Our review found that patients who develop septic shock/MOD have a 2.2 times greater risk of dying than those who do not (OR = 2.241; *p* = 0.008) (Table 2).

## 4. Discussion

Visceral disseminated varicella-zoster virus infection presents a significant diagnostic challenge due to its ability to mimic diverse clinical conditions. Early symptoms—such as abdominal pain, fever, and malaise—are nonspecific. While the vesicular rash is a hallmark sign, it is absent in approximately 5% of cases. Additionally, visceral involvement may precede cutaneous lesions, complicate early recognition, and increase the risk of severe complications [15]. Our review found that delayed diagnosis is associated with poorer clinical outcomes.

VD-VZV can involve multiple organ systems, including the lungs (pneumonia), heart (myocarditis and pericarditis), esophagus (esophagitis), liver (hepatitis and ALF), kidneys (acute kidney injury), and small intestines (enteritis). Less commonly, it is presented as pancreatitis, gastritis, or colitis. In severe cases, it may progress to septic shock or MODS, both linked to high morbidity and mortality [15,16,17]. Patients with VD-VZV who develop shock and MODS have a 2.2-fold increased risk of death compared to those without these complications.

Diagnosis is primarily clinical, supported by imaging, laboratory findings and by ruling out other common viral, bacterial and fungal pathogens. PCR testing of blood, skin, or tissue remains the gold standard. In settings where PCR is unavailable, viral culture may be used, though it is less sensitive and more labor-intensive [15,16,17]. Culturing VZV creates an infectious virus capable of airborne and contact transmission; therefore, manipulations that propagate live VZV should be performed under biosafety level (BSL)-2 containment and strict adherence to airborne/contact exposure controls.

### 4.1. Pulmonary Involvement in VD-VZV Infection

VZV is a rare cause of pneumonia, occurring during either primary infection or reactivation. Diagnosis is straightforward when typical pulmonary symptoms—fever, cough, pleuritic chest pain, chest tightness, dyspnea, tachypnea, and/or hemoptysis—coincide with a characteristic rash. However, when symptoms precede or occur without a rash, diagnosis becomes more challenging and treatment is often delayed [15,18]. In our review, 87 patients had pneumonia, making the lungs the most frequently affected internal organ in disseminated VZV. Among these, seven (8%) had no rash, and in nine (10.3%), the rash appeared after respiratory symptoms, complicating and delaying diagnosis.

VZV pneumonia is primarily diagnosed clinically, supported by imaging and molecular or serological confirmation of the active infection. Ideally, diagnosis is confirmed via PCR from a lung biopsy or bronchoalveolar lavage (BAL). In practice, it is often based on clinical presentation, presence of a rash, and timing of symptoms, after excluding more common bacterial and viral pneumonias. Co-infections have been reported, including *Staphylococcus aureus* [19], *Legionella pneumophila* [20], and in one non-HIV immunocompromised patient, fungal infection with *Pneumocystis jirovecii* alongside *Klebsiella pneumoniae*, ESBL-producing *Escherichia coli*, and *Corynebacterium striatum* [21]. In such cases, PCR testing of BAL or lung tissue is crucial due to its high sensitivity and specificity.

Common CXR findings include multiple 5–10 mm nodules, unilateral or bilateral consolidations, ill-defined or patchy nodular opacities, bronchial wall thickening, and occasionally pleural effusion or lymphadenopathy [22,23]. CT scans offer clearer detail and are preferred for evaluating parenchymal changes. CT often reveals multiple, disseminated, well-defined peripheral nodules (1–10 mm) with ground-glass halos [22,24,25,26], along with interlobular septal thickening and segmental consolidations [23].

These nodules may reflect chronic post-viral lung changes, presenting as small calcifications. Since post-inflammatory calcifications are common, distinguishing between causes can be difficult. Key differentials include other viral pneumonias (e.g., cytomegalovirus (CMV), influenza, measles), pulmonary hemosiderosis, idiopathic silicosis, and alveolar microlithiasis [25]. Calcifications from VZV are typically small (2–3 mm), well-defined, and irregularly distributed [22]. Given the radiological overlap, further clinical testing is essential.

Supportive care includes oxygen therapy, respiratory hygiene, and hydration. Intravenous acyclovir (10 mg/kg every 8 h) is the treatment of choice. Among 87 reviewed patients, 23 received steroids and 35 were treated with antibacterials for secondary bacterial pneumonia. Post-exposure prophylaxis with VZV immune globulin (VZIG) is recommended for high-risk individuals but is limited by cost and availability [27]. Three patients recovered successfully after VZIG therapy [27,28,29].

Approximately 40% of VZV pneumonia patients require mechanical ventilation. Extracorporeal membrane oxygenation (ECMO) should be considered in cases of fulminant or refractory respiratory failure [30]. Corticosteroids may reduce pulmonary inflammation, improve oxygenation, and shorten ventilation time [19,31], but prolonged use can increase the risk of opportunistic infections. In our cohort, corticosteroid use was not associated with improved survival.

### 4.2. Hepatitis and Acute Liver Failure in VD-VZV Infection

In this scoping review of 156 VD-VZV cases, hepatic involvement emerged as one of the most prevalent and clinically significant complications. Hepatitis occurred in 44.2% of cases, and ALF in 16.7%. Both were significantly more common among patients who died, with ALF independently predicting mortality in multivariate analysis (*p* = 0.039). These findings are consistent with prior reports highlighting hepatic involvement as a critical prognostic factor in disseminated VZV infection [32,33].

The mechanisms of VZV-induced liver injury are likely multifactorial, involving direct viral cytopathy and immune-mediated hepatocyte damage. Immunosuppressed patients, with impaired antiviral defenses, are particularly vulnerable to extensive viral replication and multi-organ dissemination, including hepatic involvement [1,13]. In our review, positive liver biopsy findings were significantly more frequent among fatal cases (*p* = 0.023), further underscoring the liver’s susceptibility in severe forms of VZV.

Park et al. described a kidney transplant recipient with fatal myocarditis and fulminant hepatitis despite appropriate antiviral therapy [17], while Fang et al. reported fatal hepatitis in an immunocompetent adult [34]. These cases, along with our findings, indicate that, while immunosuppression increases risk, fulminant hepatitis can occur even in non-immunocompromised individuals.

Laboratory abnormalities—elevated AST, ALT, and INR—were significantly associated with mortality (*p* < 0.001 and *p* = 0.004), supporting their role as prognostic markers. These results align with Hsing et al., who emphasized early recognition of hepatic dysfunction in severe cases of VZV infection [35].

In some cases, liver injury preceded the appearance of the characteristic vesicular rash. The rash was absent in 5% of the entire cohort but was absent in 10% of fatal cases, which led to delayed diagnosis and treatment. Delayed diagnosis was significantly more common in deceased patients (*p* = 0.043), highlighting the importance of early recognition in patients with unexplained acute liver dysfunction. Although 91% of patients received antiviral therapy, the high mortality in those with hepatic involvement suggests that treatment delay is a major contributor to poor outcomes.

### 4.3. Cardiac Complications in VD-VZV Infection

Cardiac complications of VD-VZV infection include myocarditis, pericarditis, perimyocarditis, and, rarely, endocarditis. Although VZV exhibits cardiotropism, its affinity for cardiac tissue is generally lower than that of other viruses, such as coxsackievirus, enterovirus, adenovirus, influenza virus, and other *Herpesviridae* family members. Cardiac involvement occurs in approximately 16% of disseminated VZV cases, compared to 56% and 44% for pulmonary and hepatic involvement, respectively. Cardiac complications may develop during either primary infection or viral reactivation. Myopericarditis is the most frequently observed manifestation, presenting as either isolated cardiac involvement or as part of a multisystem process [17,27,36,37,38,39,40,41,42,43,44,45,46,47,48,49,50,51,52,53]. Endocarditis is exceedingly rare, with only isolated case reports—such as the case described by Chen involving bicuspid valve endocarditis and extensive paravalvular invasion [37].

Clinical presentation may include chest pain, tachycardia, and hypotension, often preceding the appearance of the characteristic vesicular rash, thereby complicating early diagnosis. Rare manifestations include syncope due to malignant arrhythmias [38] or sudden infant death [39]. VZV-associated myopericarditis may mimic acute coronary syndrome [40,53], present with pericardial tamponade [54], or cause multiorgan failure resembling catastrophic antiphospholipid syndrome [41].

Diagnosis is typically established by detection of varicella-specific IgM antibodies or VZV DNA via PCR, alongside clinical signs of cardiac involvement and demonstrating a consistent temporal association between the rash (when present) and the onset of cardiac symptoms. Patients frequently present with fever, rash, elevated cardiac biomarkers, and tachycardia. Pericardial effusion may be observed in cases of pericarditis [54]. Electrocardiographic findings are nonspecific but may include sinus tachycardia, T-wave abnormalities, or changes suggestive of pericarditis, such as diffuse ST-segment elevation and PR depression. Echocardiography often reveals reduced left ventricular ejection fraction, which typically improves with antiviral therapy. Cardiac magnetic resonance imaging (MRI) is the most sensitive non-invasive modality for diagnosing myocarditis, though it does not distinguish between viral etiologies and may be limited by cost and availability. Endomyocardial biopsy typically reveals lymphocytic myocarditis—the most common histopathological pattern in viral myocarditis—including VZV-associated cases. Characteristic findings include lymphocytic infiltrates with myocyte necrosis, confirmed by immunohistochemistry and molecular detection of VZV DNA [36,37,38,39,40,41,42,43,44,45,46,47,48,49,50,51,52,53].

Standard treatment consists of intravenous acyclovir for 7–10 days, which usually results in clinical improvement. Adjunctive therapies such as IVIG and corticosteroids have been employed with variable success. In cases of isolated pericarditis, nonsteroidal anti-inflammatory drugs (NSAIDs) and colchicine are the primary treatments. However, NSAIDs are contraindicated in isolated myocarditis due to the potential exacerbation of viral replication and myocardial injury. Patients are generally advised to refrain from strenuous physical activity for 4–6 weeks, although this recommendation is based on expert consensus rather than randomized controlled trials.

Prognosis is generally worse in immunocompromised individuals, in part due to atypical presentations and diagnostic delays. The estimated mortality rate for VZV-related myopericarditis is approximately 30%, substantially higher than that associated with other viral etiologies [55]. However, due to the rarity of this complication, large-scale epidemiological data remain limited.

### 4.4. Gastrointestinal Tract Involvement in VD-VZV Infection

Gastrointestinal (GI) involvement in VD-VZV infection can manifest as esophagitis, gastritis, enteritis, or colitis. Isolated VZV infection distal to the gastric cardia is rare but has been sporadically reported. In most cases, GI tract involvement occurs as part of disseminated disease, with esophageal and small intestinal lesions reported more frequently than gastric or colonic involvement [23]. This review identified 32 cases of gastrointestinal (GI) involvement among patients with VD-VZV infection, representing 20.5% of the total cases analyzed. Considering the esophagus, stomach, and intestines specifically, there was a total of 43 affected organs reported among this subgroup. Intestinal infection was the most common, occurring in 56.3% of patients with GI involvement, followed by gastric involvement in 50% and esophageal involvement in 28.1% of cases. Although not independently associated with increased mortality (*p* = 0.583), intestinal involvement frequently co-occurred with other visceral complications, indicating it may be part of a more extensive disseminated disease process.

VZV is likely to affect the GI tract through direct mucosal infection and involvement of the enteric nervous system (ENS). Hematogenous spread or reactivation in enteric ganglia may lead to inflammation, ulceration, and mucosal ischemia. The small intestine may be particularly vulnerable due to its dense ENS and abundant lymphoid tissue [23]. Intestinal VZV infection is associated with severe abdominal pain, bloating, diarrhea, and gastrointestinal bleeding. It is a rare but critical differential diagnosis in severe abdominal pain and is often overlooked due to its atypical presentation and low initial clinical suspicion [56,57,58,59,60,61,62,63,64,65,66].

For suspected GI tract involvement, initial evaluation with contrast-enhanced abdominal CT is appropriate. Findings that support inflammation include fat stranding, segmental bowel wall thickening, mucosal enhancement, and signs of perforation [57,58,64,67,68,69,70]. Gastric involvement may present with epigastric pain, early satiety, nausea, vomiting, and occasionally hematemesis. Esophageal VZV typically presents with retrosternal pain, odynophagia, and dysphagia. For lesions above the ligament of Treitz, an esophagogastroduodenoscopy (EGD) with a biopsy is the preferred diagnostic procedure. Typical endoscopic findings include erythematous ulcers, flat or raised nodules, and fibrinous exudates. Histopathological features suggestive of VZV include ballooning degeneration, multinucleated giant cells, and Cowdry type A bodies [66,71]. Among 20 patients with upper GI involvement, an endoscopic evaluation with a biopsy was performed in 19 cases, yielding positive findings in 94.7%, underscoring the diagnostic value of endoscopy in suspected upper GI VZV disease.

In the reviewed cohort, all patients with presumable colitis were diagnosed based on the clinical presentation of abdominal pain, fever, diarrhea, and the typical rash temporally associated with evidence of visceral dissemination and abdominal CT findings confirming colitis in the appropriate clinical setting. Diagnosis can be challenging, especially when a typical dermatomal rash is absent or the involvement is multifocal.

In this cohort, three patients had no known comorbidities at the time of diagnosis; in each of these cases, diagnosis was delayed by more than 24 h after initial physician contact, yet all three patients survived, suggesting a potentially favorable prognosis in immunocompetent individuals despite diagnostic delay [72,73,74]. Visceral VZV infection has a high potential to mimic other conditions, contributing to frequent diagnostic delays and clinical uncertainty. VZV esophagitis may mimic acute coronary syndrome [24], while VZV enteritis can resemble other causes of acute abdomen [22,64,65]. One case presented with visceral VZV symptoms preceding any skin lesions, highlighting the potential for atypical clinical manifestations [75]. Another patient experienced a 10-day delay in diagnosis following the onset of cutaneous symptoms but showed rapid improvement with high-dose acyclovir therapy, supporting the potential benefit of initiating antiviral treatment beyond the conventional 72 h window in complicated or atypical cases [68].

In contrast, one immunocompromised patient with mucosal ulcers involving the oral cavity, esophagus, and duodenum experienced clinical deterioration upon transitioning from effective high-dose intravenous acyclovir to suppressive valacyclovir therapy, with recurrent symptoms and eventual death. This case illustrates how immunocompromised states may pose ongoing therapeutic challenges even in patients initially responsive to antiviral treatment [76]. Furthermore, clinicians should consider impaired absorption of orally administered medications in cases of extensive GI involvement. In such cases, patients would complete treatment with the intravenous formulation of acyclovir. Finally, one patient developed fatal visceral VZV infection without any skin rash; diagnosis was confirmed postmortem nine days after symptom onset, further emphasizing the diagnostic complexity of atypical presentations [77].

As for the diagnosis of other internal organs, PCR testing of biopsy specimens remains the diagnostic gold standard for detecting VZV DNA with high sensitivity and specificity [25,26]. Serological testing for VZV-specific IgM antibodies can aid in the diagnosis; however, its clinical utility is limited due to variable sensitivity and specificity, with false positives and delayed seroconversion [35,78,79,80,81]. Prompt and accurate diagnosis relies on clinical judgment and a thorough assessment of the patient’s overall presentation to avoid unnecessary investigations and delays.

Management involves the early initiation of intravenous (IV) acyclovir, which remains the first-line antiviral treatment [19]. In cases of acyclovir resistance or severe infection, alternative agents, such as foscarnet or cidofovir, may be considered [31,82]. Symptomatic management includes analgesics, antacids, H2-receptor antagonists, and proton pump inhibitors to relieve esophageal and gastric discomfort. Nutritional support and hydration are crucial to prevent complications and improve clinical outcomes [58,75,83]. Parenteral nutrition may be indicated when oral intake is significantly compromised. Modification of immunosuppressive therapy should be considered when possible. In cases with severe complications, such as transmural necrosis, hemorrhage, or perforation, surgical intervention may be necessary.

Our findings suggest that gastrointestinal symptoms in patients with risk factors for VZV should prompt an evaluation for visceral dissemination, particularly when accompanied by systemic signs of infection and poorly differentiated abdominal pain. While not independently predictive of mortality, intestinal involvement may contribute to delayed diagnosis and increased diagnostic uncertainty, especially in immunosuppressed or atypically presenting patients.

### 4.5. Pancreatic and Splenic Involvement in VD-VZV Infection

The pancreas and spleen are rare visceral organs affected by VZV infection, either primary or reactivation. Of the 156 individual cases included in this research, pancreatic-related manifestations of disseminated VZV were identified in 17 cases, while only 5 patients had splenic involvement.

Most reported cases occurred in patients who were organ transplant recipients, had hematologic malignancies, or had HIV/AIDS. All patients with pancreatic involvement were immunocompromised: seven had hematologic malignancies, four had undergone recent transplantation (within one year), one had HIV/AIDS, and the remaining were on immunosuppressive therapy for autoimmune or other conditions [16,32,57,70,75,76,77,84,85,86,87,88,89,90,91,92]. Among splenic cases, one patient was immunocompetent, while the rest were immunocompromised [36,77,93,94,95].

In all reported cases, pancreatic involvement occurred alongside other organ involvement or as part of MODS [18,96,97]. Abdominal ultrasound is often inconclusive due to its limited ability to visualize the retroperitoneal pancreas. In contrast, abdominal CT and MRI provide greater sensitivity for detecting features of acute pancreatitis. Imaging typically shows pancreatic enlargement, peripancreatic edema and inflammation, peripancreatic fat stranding, and fluid collections.

Biopsy and histopathological analysis were performed in only one deceased patient during autopsy, revealing intranuclear inclusion bodies and multinucleated giant cells indicative of VZV infection in the pancreas, spleen, and other organs [72]. Biopsy of the pancreas is technically difficult and not routinely performed in clinical practice. Instead, the diagnosis of VZV pancreatitis is based on radiologic evidence, clinical presentation, and laboratory findings within the appropriate clinical context, particularly when there is a consistent temporal association between the onset of pancreatitis and the presence of disseminated VZV infection. Confirmation of disseminated VZV infection is achieved via polymerase chain reaction (PCR) testing of skin lesion swabs or blood samples.

Management includes supportive care with fluid resuscitation and pain control using agents such as acetaminophen, NSAIDs, and, for severe pain, opioids. Of 17 patients with pancreatitis, 12 survived and 5 died (Table 1), reflecting a nearly 30% mortality rate—substantially higher than in other cases of viral pancreatitis. None of the survivors developed long-term complications, such as diabetes or exocrine pancreatic insufficiency.

The spleen is the rarest visceral organ affected in disseminated VZV infection. Splenic involvement typically presents as splenomegaly and is always accompanied by involvement of other organs, most notably the liver, which was affected in all reported cases [36,77,93,94,95]. Clinical manifestations are often nonspecific, and splenic abnormalities are usually identified incidentally during evaluation of other organ systems. Ultrasound and CT imaging have been used to detect splenomegaly [93,94]. Abdominal CT scans may also reveal multiple hypodense lesions in the spleen [95]. As with pancreatic involvement, diagnosis of disseminated VZV infection is confirmed via PCR testing of skin lesion swabs or blood samples. Spleen biopsy is not routinely performed. In some cases, such as the one described by Arakawa et al., splenic involvement occurs as part of widespread multiorgan infection. In that instance, typical cutaneous manifestations were absent. The patient presented with acute abdominal symptoms, thrombocytopenia, and hepatic failure. VZV infection was not suspected clinically and was only diagnosed postmortem through immunohistochemical and molecular evidence of viral presence in the pancreas, spleen, liver, kidneys, and other organs [72].

Unlike in some other infectious diseases, such as EBV infection, babesiosis, and malaria, where the spleen can be affected by splenic infarcts and rupture, no such occurrence has been reported in disseminated VZV infection [98,99,100].

### 4.6. Kidney Involvement in VD-VZV Infection

In our systematic review, renal involvement was noted in approximately 11% of patients. Though it is less frequently reported than hepatic or pulmonary complications, kidney involvement had significant prognostic implications. Patients with acute kidney injury (AKI) had a higher mortality rate (*p* = 0.049), suggesting that renal impairment may serve as an indicator of disease severity. The pathophysiology of VZV-related kidney injury remains incompletely understood but may include direct viral invasion, immune-mediated damage, or acute tubular necrosis resulting from systemic inflammation and hemodynamic instability. Nephrotoxicity associated with antiviral agents, particularly intravenous acyclovir, may also contribute to renal injury in the setting of VZV infection [18,88].

Previous case studies have highlighted the renal tropism of VZV in both immunocompetent and immunocompromised populations. Premužić et al. described a case of AKI as the sole manifestation of VZV in an immunocompetent adult, emphasizing that renal involvement can occur independently of other organ dysfunction [97]. Similarly, Yamada et al. reported a pediatric transplant recipient with VD-VZV who developed renal failure as part of a broader multi-organ failure syndrome [101].

In our cohort, kidney involvement was often associated with features of septic shock or MODS, both of which were also significant predictors of mortality. These findings suggest that renal involvement in VD-VZV may not only reflect direct organ damage but also serve as a marker of systemic disease progression. Early identification of renal dysfunction, coupled with appropriate fluid management and careful monitoring of nephrotoxic therapies, may be critical to improving outcomes in affected patients.

### 4.7. Role of Vaccination

The efficacy of the varicella-zoster vaccine in preventing disseminated VZV can be presumed from its success in preventing herpes zoster (shingles) and severe complications. The recombinant zoster vaccine (RZV—2 dose vaccine) yields 73–86% effectiveness against herpes zoster in multiple real-world studies with very little wanning over 4 years in both immunocompetent and immunocompromised adults. The live attenuated zoster vaccine demonstrated 46–51% efficacy in comparison. The CDC recommends two doses of RZV for adults aged >50 years and for immunocompromised adults aged >19 years [177,178,179]. However, even in vaccinated individuals lethal outcomes have been reported due to diagnostic and therapeutic delays [180].

Limitations of this scoping review are few. Firstly, due to the nature of this methodology, some high-quality reports may have been omitted for not meeting the pre-selection criteria. Articles not published in English were excluded, introducing potential language bias. There is also a possibility of publication bias, as authors may be more likely to report cases with poor outcomes or more severe clinical presentations. Secondly, a limitation of this review, inherent to the nature of its methodology—relying on data from published case reports—is the lack of complete information on patients’ vaccination status. This limitation prevented us from analyzing the impact of vaccinations on outcomes in patients with disseminated infection. Furthermore, the vast majority of cases did not specify whether the infection was primary or secondary (i.e., reactivation), nor the viral strain. Finally, while each included case met predefined diagnostic criteria and was carefully reviewed, not all reported cases had tissue biopsy or PCR confirmation—reflecting the challenges encountered in real-world clinical practice.

## 5. Conclusions

Visceral (internal) organ involvement in disseminated VZV infection is associated with high mortality and is often challenging to diagnose promptly. Immunocompromised patients tend to present with more severe clinical manifestations and frequently experience delayed diagnoses due to atypical disease presentations. Patients who are immunocompromised—particularly those receiving immunosuppressive therapy for autoimmune diseases—are especially vulnerable to poor outcomes. The lungs, liver, heart, and kidneys are the most affected organs. Mortality is strongly associated with delayed diagnosis and treatment, the development of shock, multiorgan failure, and acute liver failure.

While tissue biopsy and PCR are the gold standard for diagnosis, it is often technically difficult, associated with procedural risks and clinically impractical. Therefore, diagnosis is typically confirmed through clinical evidence of disseminated disease, positive PCR results from blood or vesicular skin lesions, and confirmation of internal organ involvement via laboratory markers or imaging studies. Treatment includes supportive care, prevention of superimposed bacterial and fungal infections, and antiviral therapy with acyclovir. Although steroidsand IVIG have been used as adjunctive treatment, their effectiveness remains inconclusive.

There is a pressing need for multicenter, prospective studies to better characterize internal organ involvement in disseminated VZV, to develop more sensitive and practical diagnostic tools, and to improve the timeliness of diagnosis—ultimately aiming to reduce the unacceptably high mortality in this vulnerable patient population.

## Figures and Tables

**Table 1:** Socio-demographic, clinical, diagnostic, and treatment characteristics of alive and deceased patients with VD-VZV infection.

Variables	Alive (*n* = 117, 75.0%)		Died (*n* = 39, 25.0%)		Total (*n* = 156, 100%)		*p*
	N	%	n	%	n	%	
**Gender**							
Male	65	55.6	19	48.7	84	53.8	0.458 *
**Age** (M ± SD)	41.56 ± 19.23		44.63 ± 25.02		42.32 ± 20.77		0.432 **
Children (2–18 years old)	16	13.8	5	13.2	21	13.6	0.921 *
Adults (19–90 years old)	100	86.2	33	86.8	133	86.4	
**Comorbidities**	69	59.0	33	84.6	102	65.4	**0.004** *
Cancer on chemotherapy	20	17.1	6	15.4	26	16.7	0.804 *
Autoimmune disease on IST	16	13.7	10	25.6	26	16.7	**0.048** *
Transplant (less than 1 year)	16	13.7	6	15.4	22	14.1	0.791 *
T2D	8	6.8	1	2.6	9	5.8	0.322 *
Corticosteroids	6	5.1	4	10.3	10	6.4	0.257 *
HIV/AIDS	5	4.3	1	2.6	6	3.8	0.631 *
Immunosuppressants	3	2.6	1	2.6	4	2.6	1.000 *
**Multiple organ involvement**	50	42.7	22	56.4	72	46.1	0.139 *
Skin	113	96.6	35	89.7	148	94.9	0.094 *
Lungs	66	56.4	21	53.8	87	55.8	0.780 *
Liver	46	39.3	23	59.0	69	44.2	**0.032** *
Septic shock or multiple organ dysfunction	43	36.8	29	74.4	72	46.2	**<0.001** *
Heart	18	15.4	7	17.9	25	16.0	0.705 *
Intestines	13	11.1	5	12.8	18	11.5	0.772 *
Pancreas	12	10.3	5	12.8	17	10.9	0.656 *
Kidneys	10	8.5	7	17.9	17	10.9	**0.049** *
Stomach	14	12.0	2	5.1	16	10.3	0.223 *
Esophagus	7	6.0	2	5.1	9	5.8	0.843 *
Spleen	2	1.7	3	7.7	5	3.2	0.066 *
Gallbladder	3	2.6	1	2.6	4	2.6	1.000 *
Peritoneum	1	0.9	1	2.6	2	1.3	0.411 *
Pharynx	0	0.0	1	2.6	1	0.6	0.082 *
Bladder	0	0.0	1	2.6	1	0.6	0.082 *
**Acute liver failure**	12	10.3	14	35.9	26	16.7	**<0.001** *
**Biopsy performed**	52	44.4	20	51.3	72	46.2	0.458 *
Positive biopsy finding	49	94.2	18	90.0	67	93.1	0.527 *
Skin	38	32.5	13	33.3	51	32.7	0.922 *
Esophagus	2	1.7	1	2.6	3	1.9	0.736 *
Heart	2	1.7	1	2.6	3	1.9	0.736 *
Lungs	3	2.6	5	12.8	8	5.1	**0.012** *
Stomach	11	9.4	2	5.1	13	8.3	0.403 *
Intestines	3	2.6	2	5.1	5	3.2	0.431 *
Liver	4	3.4	5	12.8	9	5.8	**0.029** *
Pancreas	0	0.0	1	2.6	1	0.6	0.082 *
Kidneys	1	0.8	0	0.0	1	0.6	0.562 *
**PCR VZV performed**	50	42.7	25	64.1	75	48.1	**0.021** *
Positive PCR VZV finding	48	96.0	25	100.0	73	97.3	0.311 *
**VZV IgM and IgG positivity**							
VZV IgM positive	40	75.5	8	57.1	48	71.6	0.176 *
VZV IgG positive	42	70.0	8	42.1	50	63.3	**0.028** *
**VZV vaccination status**							
Negative	17	14.5	8	20.5	25	16.0	0.732 *
Positive	16	13.7	4	10.3	20	12.8	0.889 *
Not reported	84	71.8	27	69.2	111	71.2	0.901 *
**Laboratory analyses** (Mdn/IQR)							
AST (U/L)	68.50/12.35		1903.40/902.00		1131.00/962.50		**<0.001** *^#^*
ALT (U/L)	111.00/21.50		1227.50/412.50		732.50/438.40		**<0.001** *^#^*
ALP (IU/L)	244.00/55.50		502.0/351.50		221.50/328.00		0.391 *^#^*
Total bilirubin (mg/dL)	0.62/0.29		4.00/1.91		1.61/5.18		0.886 *^#^*
INR	1.09/0.86		2.48/1.09		1.82/1.39		**0.004 *^#^***
**Treatment**							
Antivirals	109	93.2	33	84.6	142	91.0	0.106 *
Antibacterial antibiotics	43	36.8	17	43.6	60	38.5	0.447 *
Antivirals and antibacterial	41	35.0	16	41.0	57	36.5	0.502 *
Steroids	25	21.4	10	25.6	35	22.4	0.580 *

PCR—polymerase chain reaction, VZV—varicella-zoster virus, AST—aspartate aminotransferase, ALT—alanine aminotransferase, ALP—alkaline phosphatase, INR—international normalized ratio, T2D—type 2 diabetes; IST—immunosuppressive therapy, HIV—human immunodeficiency virus, AIDS—acquired immune deficiency syndrome, Mdn—median, IQR—interquartile range, M—mean ± SD—standard deviation, *p*—statistical significance was measured by * χ^2^—chi square test, ** Independent *t* test and ^#^ Mann-Whitney U test, significant values are bolded.

**Table 2:** Multivariate analysis of variables and their association with mortality in our systematic review.

Variables	B	OR	95% CI	*p*
Comorbidities	1.272	1.419	0.609–4.230	**<0.001**
Autoimmune disease on IST	0.058	0.323	0.203–0.994	0.806
Other comorbidities	0.299	0.661	0.490–2.005	0.229
Septic shock or multiple organ dysfunction	2.632	2.241	1.190–6.007	**0.008**
Liver involvement	0.135	0.991	0.563–1.588	0.272
Kidney involvement	0.153	0.229	0.092–0.831	0.779
Acute liver failure	1.582	0.831	0.661–2.690	**0.039**
Performance of VZV PCR	0.128	0.378	0.109–2.103	0.886
Positive VZV IgG	−0.190	0.813	0.193–3.407	0.821
AST	0.138	1.149	0.845–4.373	0.353
ALT	0.153	0.669	0.306–1.839	0.643
INR	2.003	7.932	1.217–72.203	0.390
Constant	4.201	1.449	/	0.203

IST—immunosuppressive therapy, PCR—polymerase chain reaction, VZV—varicella-zoster virus, AST—aspartate aminotransferase, ALT—alanine aminotransferase, INR—international normalized ratio, CT—computerized tomography, B—unstandardized regression coefficient; OR—odds ratio; 95% CI—confidence interval, *p*—statistical significance; significant values are bolded.

## Data Availability

The original contributions presented in this study are included in the article. Further inquiries can be directed to the  corresponding author.
